# The SOR SAVOIR PATIENT project–an evidence-based patient information and education project

**DOI:** 10.1038/sj.bjc.6601093

**Published:** 2003-08-15

**Authors:** B Fervers, L Leichtnam-Dugarin, J Carretier, V Delavigne, H Hoarau, S Brusco, T Philip

**Affiliations:** 1 FNCLCC, Paris, France; 2 Centre Leon Bérard, Lyon, France

The management of patients with cancer has evolved over recent years due to the development of the concept of ‘evidence-based medicine’ and a change in the patient–physician relationship. Evidence-based medicine assigns a greater importance to the results from clinical research in the medical decision-making process than was assigned in the past. The change in the patient–physician relationship has arisen from a request from patients for better information concerning their illness and its management, and for a greater level of involvement in the treatment decisions.

Information and decision-making are both aspects of a patient-centred approach to cancer care (this can also be defined as patient education) and are integral parts of the health-care process, although there is some controversy about the compatibility of these concepts (Bensing, 2000; Straus and McAlister, 2000; Stewart, 2001).

## ROLE OF PATIENT INFORMATION

The results from many studies have highlighted that patients with cancer and their families express an important need for information. Patient's satisfaction with their management is closely related to the response to this need. The patients' understanding of their illness can help them to accept their condition and to live with their illness and participate in the care process. The absence of information seems to play an important role in the difficulties of psychological adaptation associated with being informed about the diagnosis and with the treatment (Leydon *et al*, 2000). Several reports have identified patient information as a major quality indicator in the overall management of patients, for example, the French Agency for Accreditation and Evaluation in Health (ANAES) defines patient information and education as a criteria of quality in its accreditation system. In addition, in many countries the patients' rights for information and the physicians' obligations are enforced by law.

Nonetheless, the patients' need to be informed about the diagnosis, the treatment and the prognosis is often under-estimated by physicians (Ong *et al*, 1995; Vennin *et al*, 1995; Butow *et al*, 1997; Degner *et al*, 1997).

Patients' information needs (type and amount of detail) differ between individuals and change over the various stages of the care process (i.e. diagnosis, treatment, follow-up, rehabilitation, remission, recurrent and advanced disease) and the patients' acceptation of the illness (Demma *et al*, 1999; Leydon *et al*, 2000). In addition, several studies have shown that the patients who want to be informed about the disease and treatment do not always want to participate in the treatment decision (Cassileth *et al*, 1980; Sutherland *et al*, 1989; Blanchard *et al*, 1990; Moumjid-Ferdjaoui and Carrere, 2000). Some patients want to know why a particular treatment is chosen, but they do not want to participate in the choice.

The discrepancies between physicians and patients' perception of information needs, and the length of consultation, which is often insufficient, are important barriers to providing patients with appropriate information within the clinical setting. Many patients actively search for information on their illness and its treatment to satisfy their need for information. For example, in a study of 191 patients with cancer in Canada, 71% reported that they had actively searched for information and 50% had used Internet. However, the physician remained the main information source for 83% of these patients (Chen and Siu, 2001).

## PATIENT INFORMATION MATERIALS

The increased demand of patients with cancer for information should be met with more complete information on the benefits and also the side effects of treatment than has been previously available (Coulter, 1998). However, in oncology, the treatment schemas are often complicated, and the specialised technical language used is difficult to understand for an untrained person (Hadlow and Pitts, 1991). This increased demand for appropriate information has led to an increased development of information materials.

Unfortunately, much of the information available is inconsistent with the scientific literature and does not meet the needs of the patients (Coulter *et al*, 1999). They often give an overoptimistic view of the treatment benefits and side effects, and they fail to mention the primary information sources on which the information is based. An analysis of more than 100 patient information booklets performed by the FNCLCC found similar results (unpublished). In addition, there was no clear mention of the involvement of patients in the preparation of the booklets.

## THE SOR SAVOIR PATIENT PROJECT

In response to the evolution of the information-seeking behaviour of patients, the French National Federation of Cancer Centres (Federation Nationale des Centres de Lutte Contre le Cancer; FNCLCC) initiated the SOR SAVOIR PATIENT project in 1998. This is a programme for the development of evidence-based patient information and it falls within the framework of patient education. Patient education involves a series of structured activities, which are integrated into the health-care process, with the aim of improving patients' understanding of cancer treatment and their management of their disease. Patient education also aims to provide psychological support, facilitate physician–patient relationships and favour patients' involvement in treatment decisions. One important element is the development of information documents adapted to the needs and experiences of the patients and their families.

### Objectives

The objectives of the SOR SAVOIR PATIENT project are:

- to enable patients to understand the fundamental medical knowledge about their illness and its management;
- to improve the participation of patients in the management of their illness and in treatment decisions;
- to improve the patients' recognition and management of the consequences and side effects of their illness and its treatment;
- to facilitate the dialogue between the patient and the physician.

### Methodology

The medical information on which the patient information produced by this project is based on clinical practice guidelines produced by the FNCLCC for health-care specialists (Fervers *et al*, 2001). These guidelines are produced in the setting of a national collaboration between the FNCLCC and the 20 regional cancer centres in France, and the active participation of specialists from public and private sectors, the French Oncology Federation of university hospitals and many learned societies who form a multidisciplinary working group for each specific guideline.

**Figure 1:**
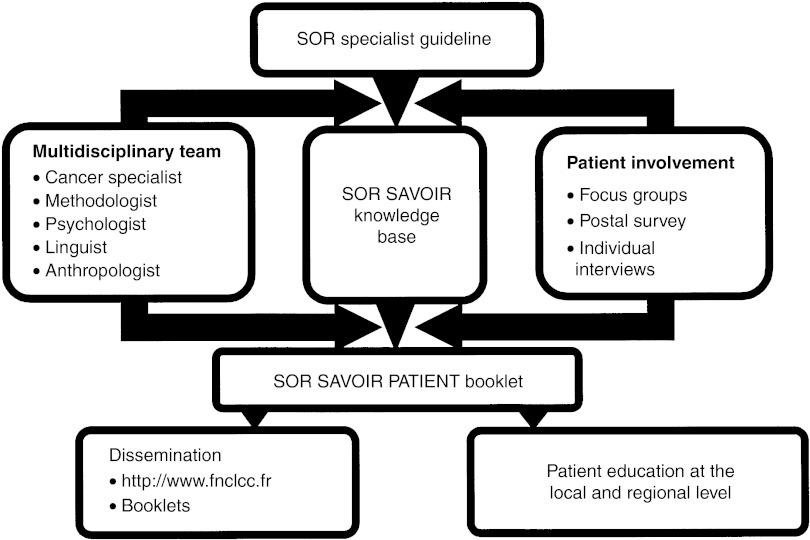
Outline of the methodology used in the SOR SAVOIR Patient project

The development of the SOR SAVOIR PATIENT guides involves the following steps (Figure 1

).

- The relevant specialist SOR guideline is used as the primary information source, and it is ‘translated’ into simple language by a multidisciplinary team, including methodologists, a linguist, a health anthropologist and oncology experts. This translation aims to reword the scientific information to satisfy specified readability criteria and the assumed knowledge level of the reader. The end product is a knowledge base which is used to produce the final patient information.
- Once established, the knowledge base is adapted to the expressed needs of the patients and their families. Patients and their family members are actively involved throughout the process to identify specific information needs and adjust the presentation of the information to the patients' perception, experience and their level of understanding. This is facilitated throughout the development process using three complementary techniques: questionnaires, focus groups and individual interviews.
- A professional working group is established (oncologists, organ specialists and other health professions concerned by the subject). This group is consulted throughout the development steps which produce the knowledge base and the SOR SAVOIR booklets. This working group validates the scientific and medical content of the information in the knowledge base and the booklets.

The final SOR SAVOIR PATIENT booklets are composed of sections (e.g. general information, diagnosis, treatment) which can be read independently, depending on the patients' specific information needs. Summaries are provided at the end of each section, and these contain key information, identified by the participating patients. There is a glossary at the end of each guide, explaining any medical and/or technical terms used in the guide. When relevant, there are practical information sheets providing information about how different examinations are performed. In the Appendix we present the translation from French to English (without the involvement of patients) of extracts from two of these practical information sheets. In addition to the information given in these examples, practical information regarding administrative and organisational matters is also given. However, since this information is specific to the French setting, we did not include it in the translated extracts.

### Dissemination and implementation of the SOR SAVOIR PATIENT booklets

**Figure 2:**
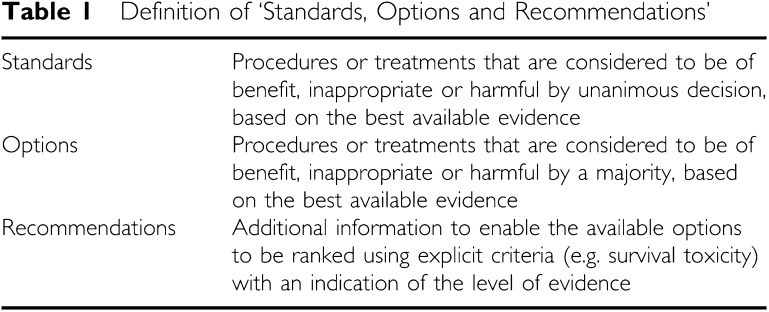
The SOR SAVOIR Patient booklets produced up to 2002

The SOR SAVOIR PATIENT booklets are published on paper (Figure 2

), and are available free-of-charge on the FNCLCC website (http://www.fnclcc.fr). Shortly, the information will be available on the site in a hyperlink version. In the paper versions, attention is paid to the presentation of the information, in particular, the colours, the font (type and size) and also the page layout. Patients are also involved in this step.

### Production up to 2002

Based on this methodology, five patient information guides (nonmetastatic breast cancer (2000, updated in 2002), neuroblastoma (2000), ovarian cancer (2002) and family risk of breast cancer and ovarian cancer (2002)) have been published up to the end of 2002.

The publication of seven other booklets is planned for 2003: understanding nonmetastatic non-small cell lung cancer; understanding cancer of the prostate; understanding osteosarcoma; understanding chemotherapy; understanding radiotherapy; understanding medical imaging (including positron emission tomography scanning); finding your way around social services and administration for patients with cancer and their families.

So far, over 100 patients have participated in the elaboration of the various SOR SAVOIR PATIENT booklets. Over 33 000 copies of the initial breast cancer booklet have been distributed from its publication up to November 2002. In 2001, this booklet was awarded a prize from the French medical journal ‘La Revue Prescrire’ in recognition of its quality. In response to the changes in available evidence, the booklet has been updated and was published in December 2002.

## PATIENT INFORMATION IN THE SETTING OF THE PHYSICIAN–PATIENT RELATIONSHIP

Written information will never replace verbal communication in the physician–patient relationship since it cannot respond to individual fundamental needs, such as being listened to, or meet the patient's demand for individualised information and psychological support. Physicians, therefore, will remain the main information source for cancer patients (Chen and Siu, 2001).

The SOR SAVOIR PATIENT project aims to respond to the request from the patients for better information about their illness and its management, and to facilitate the communication of that information. The use of SOR SAVOIR PATIENT booklets during the physician–patient encounter is intended to facilitate and aid the dialogue between the patients and their physicians. This dialogue should enable the needs of the patient to be identified, and a personalised approach taken. In this setting, the booklets offer an advantage because the organisation of the information in the booklets enables the transmission of specific information, depending on the phase of the disease and the process of care (diagnosis, treatment, follow-up).

It is essential that the transmission of the information meets the needs of both the health-care professionals and the patients. Since the SOR SAVOIR PATIENT booklets aim to meet these needs, they will play a major role in informing patients and will lead to an improvement in their management. However, the impact of these booklets on patients' outcomes and physician–patient relationships will have to be assessed. More research is needed, in particular to better understand the cultural context and to identify how to overcome existing barriers.

## EUROPEAN COLLABORATION TO SHARE EXPERIENCES

The development of good-quality patient information is a major challenge in any initiative to improve the quality of patient care (Coulter, 1998). It is generally agreed by researchers in this field that the development of good-quality information material must combine both evidence-based information sources and the active participation of patients with cancer throughout the process.

This active patient involvement should ensure that their specific information needs are satisfied, that the choice of language is appropriate, and that the patient's perception of illness and treatments is taken into consideration. However, because the information needs of patients differs, it is difficult to guarantee that the participating patients are representative. The best methods for involving patients in the process also need to be explored. The development of evidence-based patient information has raised other important questions, for example, how should the probabilities associated with the benefits and harms be presented. The results from some published studies suggest that numerical risk information should be presented, but since these results are from an English-speaking setting, and are, therefore, probably culturally specific, we need to assess if they can be extrapolated to other cultural and linguistic contexts (Edwards *et al*, 2003).

Thus, there are many areas that require further research, although we do know that practice and experiences vary from one country to another. In the project to set up an International Network for Clinical Practice Guidelines in Oncology (Philip *et al*, 2003), it is planned to address these research issues. The organisational, cultural and linguistic differences will be explored in the project in collaboration with the partners from different settings.

## Appendix A. SOR-SAVOIR PATIENT–Fact sheet: breast ultrasonography

### What is breast ultrasonography?

Breast ultrasonography is a technique in which high-frequency sound (ultrasound) waves are used to produce pictures of the inside of the breast. This examination is performed by a radiologist. The pictures produced are very useful to see if the nodules detected during *clinical examination* of the breasts or on a *mammogram* are liquid or solid.

This examination is complementary to a ^*^*mammography*. Although breast ultrasonography does not replace mammography (the reference technique for breast examination), it is often sufficient on its own.

Ultrasound provides ‘real-time’ images, that is, images or pictures that can be seen immediately. It is therefore possible to compare what has been detected by hand (during the clinical examination of the breasts) with what is visible on the screen. Also, ultrasound can be used to guide a needle towards an abnormality to obtain a sample for further testing. There is no radiation risk from this examination and it is not painful.

Although mammography is considered to be the standard examination, it is not as good as ultrasonography for detecting abnormalities in the breasts of teenagers, young women and pregnant women. Breast ultrasonography is, therefore, the examination of choice for these groups of women.

#### What should be done before breast ultrasonography?

*(If the ultrasound is not performed at the same time as the mammography)*

A breast ultrasound can be performed at any time during your menstrual cycle. There is no need to fast or avoid specific foods. You should tell the radiologist if you are taking any medication.

Before coming for the examination there is no particular preparation needed. However, you should not use any cosmetic products (cream, perfume, etc.) on your skin or wear any jewellery. You will be more comfortable if you wear a loose or buttoned top and skirt or trousers, rather than a dress, since you will be asked to remove the top half of your clothes.

Bring *your previous mammograms*, *other breast radiology reports* (ultrasound, *MRI*) and also any *results from previous examinations* of your breasts (e.g. **sampling*, surgery reports) that you have. These will be useful for the radiologist who will be able to see the abnormalities that prompted your physician to send you for a breast ultrasound. If the radiologist detects an abnormality, the information in these documents may explain it, so that further examinations may be unnecessary.

It is important to inform the radiologist about any surgical interventions that you have had on your breast. Even if the scars are not visible, they can affect the ultrasound image. Also tell the radiologist if you have a breast implant.

#### What happens during the breast ultrasound examination?

On arrivalyou should report to reception and you will be asked to wait a few minutes in the waiting room. Although the staff will make every effort to ensure that you are seen on time, unexpected delays can arise and you may have to wait a while.

A **radiographer* will take your complete file containing your doctor's referral letter and all the results of your previous breast examinations and will show you to the examination room. He/she will explain what will happen during the examination.

The radiologist will ask you some questions, read the mammography report (if you have one) and the referral letter from your doctor. The radiologist will then perform a **clinical examination* of your breasts and under your arms. For the ultrasound examination, a thin coating of lubricating gel will be spread over the area to be imaged to guarantee good contact between your skin and the ultrasound probe. The gel is hypoallergenic and allergies are rarely seen. The radiologist will examine all parts of your breast(s) to find the abnormality that cannot be explained by the mammography.

The ultrasound examination usually takes about 5–10 min, but it will take a bit longer if samples are taken during the examination. The radiologist will print out pictures of what has been seen during the examination.

### Follow-up and results of a breast ultrasound examination

The radiologist will explain the results of the clinical and ultrasound examinations. However, it is not always possible to give a definite diagnosis immediately. The pictures obtained with the ultrasound enable certain abnormalities in the breast to be seen, but it is not always possible to know for certain if the abnormality is cancerous (i.e. malignant) or noncancerous (i.e. benign). This often requires additional examinations. A report of the examination will be given or sent to you. A copy of this report will also be sent to the doctor(s) of your choice. It is very important that you keep this report somewhere safe as it will be useful for your next breast examination.
Don't hesitate to ask the members of the medical team questions about anything that is worrying you

### GLOSSARY

*Clinical examination*: An examination performed by a doctor who, after having asked you questions about your illness, will examine you with a stethoscope and use his/her hands to feel any lumps.
*Magnetic resonance imaging (MRI)*: A technique that uses radio waves (identical to those used in mobile phones) to produce pictures. The radio waves make the water molecules in your body vibrate or *resonate*. The term *magnetic* signifies that a magnet is used in the process of creating the pictures.
*Mammogram*: An image of the internal breast structure produced using **mammography*.
*Mammography*: A technique that uses a small amount of X-rays to produce images of the internal breast structure. More information can be obtained by reading the fact sheet: ‘What is mammography?’.
*Radiographer*: A person who helps the radiologist during radiological examinations, and who is a qualified technician in medical imaging.
*Sampling*: The act of taking something from your body to analyse it. Blood or liquid samples are taken using a *fine needle*. Taking a sample of cells is called a *fine needle aspiration* for *cytology*. Taking a tissue sample is called a *biopsy*. Usually, a needle is used to take breast samples through the skin, after having numbed the area. The needle is guided using a radiology apparatus (ultrasound, mammography, or more rarely, *MRI*). Sometimes sampling requires surgery.

## Appendix B. SOR-SAVOIR PATIENT–Fact sheet: Preoperative breast localisation

### What is preoperative breast localisation?

**Mammography* can be used to detect abnormalities in the breast that are so small that they cannot always be felt during a clinical examination. Some of these abnormalities are difficult, or even impossible, to locate during surgery. In addition, breasts do not have the same shape when they are compressed for the **mammogram* and when you are lying on your back for surgery.

Preoperative breast localisation is used to show the surgeon where an abnormality, detected on a mammogram, is located so that it can be found more easily during an operation. It can also help to ensure that the smallest amount possible of healthy breast tissue is removed with the abnormality.

The radiologist will look at the results of any previous examinations in your notes before deciding what method to use. Sometimes localisation involves simply marking the skin with ink to show the position of the abnormality. Often, however, a marker is placed next to the abnormality inside the breast. This marker is made up of a thread (metal or nylon) with a hook-like device at the end. This device is hooked onto the abnormality that is to be removed. The whole ‘thread–hook’ marker is folded inside a needle. The needle is inserted through the skin. When it comes into contact with the abnormality, the marker is released. The radiologist may give you an injection to numb the area around where the needle is to be inserted, so that you will not feel any pain. This is all performed under radiological control (**mammography* or **ultrasound*), so that the abnormality can be located precisely.

The marker is usually inserted the day you are admitted to the hospital, if you arrive the day before your operation is planned. However, if you are admitted to the hospital on the day of your operation, the marker will be positioned just before the operation.

We can assure you that this technique will not worsen your illness, and it will not increase the likelihood of the spread of any cancer cells. Very rarely, the insertion of the marker can cause a **haematoma* or a bruise. Certain patients, who are prone to fainting when they are emotional, may suffer from a general feeling of being unwell. When you make your appointment, please mention if you think that you are likely to react in this way. We can prescribe a drug to prevent this.

### How to prepare for the preoperative breast localisation

Before coming for the examination there is no particular preparation needed. However, you should not use any cosmetic products (cream, perfume, etc.) on your skin or wear any jewellery. You will be more comfortable if you wear a loose or buttoned top and skirt or trousers, rather than a dress, since you will be asked to remove the top half of your clothes. You should also follow any advice that the surgeon may give you during your consultation. There is no need to fast if you are admitted to hospital the day before your operation. You can eat a light snack. You should tell the radiologist about any drugs you are taking when you make the appointment. If you are taking aspirin or drugs to thin your blood, the radiologist will explain what you should do to temporarily stop taking these treatments.

Bring *your previous mammograms* and *any other breast radiology reports*(*ultrasound*, *MRI*) that you have and also any *results from any other previous examinations* that you have undergone for your breasts (e.g. **samples*, surgery reports). These documents will help the radiologist to choose the most suitable technique to use.

### What happens during preoperative breast localisation?

After completing the admission formalities, a receptionist will tell you how to get to the surgical ward. You will be shown to your bed by a nurse. When it is time for your appointment you will be taken to the radiology department waiting room with your complete file. Although the staff will make every effort to ensure that you are seen on time, unexpected delays can arise and you may have to wait a while.

The ^*^*radiographer* will take your complete file containing the surgeon's letter, and all the results of your previous breast examinations. He/she will wash your breast and explain what will happen during the examination. The complete procedure takes, on average, 30 min.

*If the radiologist is going to use ultrasound*, you will be asked to remove the top half of your clothing and to lie on your back. The radiologist will then perform an ultrasound examination to re-identify the location of the abnormality and decide exactly where to insert the needle. You may be asked to place your arm over your head and the skin around your breast will be disinfected.

The radiologist will then give you an injection under your skin to numb the area around where the needle will be inserted. You will feel the numbness within a few seconds and this will last for more than 45 min. You will not feel any pain during the examination or afterwards once the effects of the injection have worn off.

The needle will be inserted towards the abnormality, guided by the image on the screen of the ultrasound machine. When the needle reaches the abnormality, the marker will be released and the needle removed. The thread will be visible on the outside of the breast. A plaster will be placed on your breast to protect the thread.

If the radiologist is going to use mammography, you will be asked to remove the top half of your clothing and to sit down. Your breast will be slightly compressed and the radiographer will take two images of the area with the abnormality at different angles to each other. These images will be used to calculate the exact position of the abnormality in your breast using a computer. Sometimes a wire mesh guide is placed on the breast to help the radiologist to guide the needle by inserting it through a hole in the mesh facing the abnormality.

The radiologist will give you an injection under your skin to numb the area around where the needle will be inserted. You will feel the numbness within a few seconds and this will last for more than 45 min. You will not feel any pain during the examination or afterwards once the effects of the injection have worn off.

The radiologist will insert the needle towards the abnormality, guided by the calculation performed by the computer. When the needle reaches the abnormality, the marker will be released and the needle removed. The thread will be visible on the outside of the breast. A plaster will be placed on your breast to protect the thread.

Immediately after positioning the marker, two new mammograms (at different angles to each other) will be taken to make sure that the marker is well positioned. These mammograms will be given to the surgeon with the radiological report.

#### Follow-up and results of preoperative breast localisation

In the hours following the insertion of the marker, please tell the nurse in your ward if you notice any bleeding under the plaster, or pain in your breast.

During the day after the insertion, rest calmly in your bed and avoid walking around the ward. Also, avoid any unnecessary effort with your arm on the side where the marker has been inserted.

Do not sleep on your stomach or on the side where the marker has been inserted. This could displace the marker in your breast.

The area on the breast through which the marker is placed is generally not the same as that used in the operation on your breast. There will be no scar at the place that the needle was inserted.

Don't hesitate to ask the members of the medical team questions about anything that is worrying you

### GLOSSARY

*Magnetic resonance imaging (MRI)*: A medical imaging technique that uses radio waves (identical to those used in mobile phones), which makes the water molecules in your body vibrate or resonate. The term magnetic signifies that a magnet is used in the process of creating the images.
*Mammogram*: An image of the internal breast structure produced using mammography.
*Mammography*: A technique that uses a small amount of X-rays to produce images of the internal breast structure. More information can be obtained by reading the fact sheet: ‘What is mammography?’.
*Radiographer*: A person who helps the radiologist during radiological examinations, and who is a qualified technician in medical imagery.
*Ultrasound*: An imaging technique in which high-frequency sound (ultrasound) waves are used to produce pictures of the inside of the breast. The ultrasound image is very useful for distinguishing between liquid and solid nodules detected during clinical breast examination or on a mammograph. This examination can provide additional information, particularly if the mammogram is difficult to interpret. Breast ultrasound will never replace mammography, because they cannot provide the information necessary to analyse certain structures, except in particular situations (e.g., pregnant women and teenagers). More information can be obtained on the fact sheet: ‘What is a breast ultrasound?’.
